# Association of Vitamin D Receptor Gene Polymorphisms with Periodontitis 

**DOI:** 10.30476/dentjods.2025.103866.2485

**Published:** 2026-03-01

**Authors:** Mohammad Kami, Marie Saghaeian Jazi, Mostafa Allahyari, Elham Fakhari

**Affiliations:** 1 Dental Research Center, Golestan University of Medical Sciences, Gorgan, Iran.; 2 Metabolic Disorders Research Center, Golestan University of Medical Sciences, Gorgan, Iran.

**Keywords:** Polymorphism, Periodontitis, Vitamin D receptor

## Abstract

**Background:**

Considering the role of vitamin D in regulation of the bone metabolism and the immune system, some have suggested the effect of vitamin D receptor gene polymorphism in susceptibility to periodontitis.

**Purpose:**

We aimed to determine the association between vitamin D receptor (VDR) gene polymorphisms *Fok1*, *Apa1*, *Bsm1*, and *Taq1 *and periodontitis.

**Materials and Method:**

In this case-control study, 51 patients with stage Ⅲ periodontitis (case group) and 51 without (control group) were enrolled. Blood samples were taken to analyze the single nucleotide polymorphism (SNP) using polymerase chain reaction-restriction fragment length polymorphism (PCR-RFLP) after DNA extraction. Finally, the data were analyzed using SPSS software version 17, SNPStats, using Chi-square and t-test.

**Results:**

The allelic frequencies of SNP1 (*TaqI*) (*p* Value=0.089), SNP2 (*ApaI*) (*p*= 0.481), SNP3 (*BsmI*) (*p*= 0.566), and SNP4 (*FokI*) (*p*= 0.658) were not different between the groups. Genotype frequencies in the four hereditary models of codominant, dominant, recessive and, overdominant were not different in any SNPs. Of 12 haplotypes identified in the study population, TCbF was most frequent in the case group and tABF in the control group (*p*> 0.05).

**Conclusion:**

No significant association was found between VDR gene polymorphisms and periodontitis.

## Introduction

Periodontal diseases are inflammatory diseases involving soft and hard tissues supporting teeth with a global prevalence of 10-15% in general population [ 1 ]. It is primarily an infectious disease of the periodontium, triggered by a specific bacterium [ 2 - 3 ]. Alveolar bone loss with pocket formation and gingival recession are observed which may induce tooth loss. Various factors, including systemic, dental, and genetic factors are involved in the disease progression and pathogenesis [ 4 ]. 

Severe forms of periodontitis involve 7-13% of adults worldwide [ 5 - 6 ] with a significant negative impact on the oral health and quality of life of the patients [ 7 ]. Genetic predisposition has been suggested as an important factor in the occurrence and development of periodontitis [ 8 ]. This association was primarily based on the increased risk of periodontitis occurrence in genetically identical monozygotic twins with greater similarities in attachment loss and probing depth (PD), compared with dizygotic twins, with an estimated heritability of about 50% [ 9 ]. Genetic polymorphisms, which refer to different forms of a single gene, are suggested as an important genetic variation that can explain the in-dividual differences in the ability of the immune system to respond to tissue injury. Accordingly, polymorphisms in cytokine genes and receptors and metabolism-related genes (that include receptors of vitamin D and calcium) have been associated with periodontal diseases [ 10 ]. 

Vitamin D (1,25 dihydroxy vitamin D3) is a fat-soluble steroid hormone with different roles in the body, including regulation of the calcium and phosphorus metabolism, and parathyroid hormone [ 11 ]. This vitamin also plays a role in the body’s immune system by inhibiting the proliferation of lymphocytes, stimulating the differentiation of monocytes, and increasing the secretion of cytokines, such as interleukins and interferons [ 12 ]. These functions are enabled by vitamin D through its receptor. The complex of vitamin D and vitamin D receptor (VDR) enters the nucleus and binds to the vitamin D response elements (VDREs) to regulate the target genes expression through its DNA binding ability and transactivation function. The VDR is coded by the VDR gene (ID:7421, Location:12q13.11) that includes 12 exons. It is expressed in the intestine, the thyroid, and the kidney [ 13 ]. At the nucleotide level, a gene that codes a specific protein may have differences in its sequence, known as single nucleotide polymorphism (SNP); generally, these polymorphisms do not change the overall form of the product significantly to form a new protein, but they influence the binding ability and other functions of that protein [ 14 ] that can justify their association with diseases, such as periodontitis [ 15 ]. The *TaqI*, *FokI*, *ApaI*, and *BsmI* are some examples of VDR gene polymorphisms. A meta-analysis of 15 studies has revealed a significant role for VDR gene in periodontitis [ 16 ]; however the frequency of VDR gene genotypes has been reported to be different based on the population’s race [ 16 ]. According to the importance of the issue and the lack of a similar study in the target population, in this study, we aimed to determine the association between VDR gene polymorphisms (*Fok1*, *Apa1*, *Bsm1*, and Taq1) and stage III of periodontitis.

## Materials and Method

In this case-control study, adult patients referred to Dental School, Golestan University of Medical Sciences, Golestan, Iran, during 2021-2022 were enrolled. The patients who were referred to the Periodontology Department and were diagnosed with stage Ⅲ of periodontitis were considered the case group, and the patients referred to other departments of the same center and did not have periodontitis were considered the control group. The sample size was calculated at 51 in each group based on the study by El-Jilani *et al*. [ 17 ], considering a confidence level of 0.95 and a study power of 0.85 using the following equation: 

$n_{1},n_{2}=\frac{{\left(Z_{1-\frac{\alpha }{2}}+P_{1-\beta }\right)}^{2}[P_{1}-(1-P_{1})+P_{2}-(1-P_{2})]}{{\left(P_{1}-P_{2}\right)}^{2}}$

All participants were selected from a single ethnicity, were at least 18 years old, and were non-smoker, non-pregnant, non-lactating, did not have systemic chronic diseases like diabetes mellitus, did not use alcohol or other substances, and did not use antibiotics or other treatments for periodontal diseases in the past 3 months. Also, the participants who used medications that influenced their periodontal condition were not enrolled in the study. The participants had at least 18 natural teeth (except the remaining roots and wisdom teeth). Eligible patients were enrolled in the study using a simple random sampling method, and the control group was matched based on the age, sex, and plaque index with the case group.

This study was approved by the Ethics Committee of Golestan University of Medical Sciences (code: IR.GOUMS.REC.1399.406). After the researcher selected the eligible participants, the study objectives were explained to them and then they were asked to read and sign the written informed consent form. 

All participants were examined by an expert clinician, who recorded the probing depth (PD), and clinical attachment loss (CAL) using William’s probe in six dental regions (mesiobuccal, mid-buccal, distobuccal, mesiolingual, lingual, and distolingual). The O’Leary’s plaque index was used to record the plaque index and homogenize oral hygiene in the study population [ 18 ]. Diagnosis of stage Ⅲ periodontitis was made by CAL≥ 5mm and PD≥ 6mm in more than 30% of regions and loss of four or fewer teeth due to periodontitis. The control group had less than 10% sites with bleeding of probing, all sites with probing depths ≤ 3 mm and absence of CAL and bone loss [ 19 ]. 

For analyzing single nucleotide polymorphism, 5 mL of blood samples were obtained from patients’ anti-cubital fossa. Then the samples were transferred to tubes containing EDTA (as anti-coagulant) and kept in the fridge at -20°C. The DNA was extracted using Ron’s Blood and cell DNA mini kit (BIRON GmbH, Germany) and based on the manufacturer’s instructions. The extracted DNA was amplified using Taq DNA polymerase red master mix from Amplicon (Denmark) and specific primers carried out in ABI thermal cycler. The thermal cycler program for *Taq1 *and *ApaI* was as follows: 95°C for 5min as initial denaturation, followed by 35 cycles of denaturation at 95°C for 1min and annealing at 66°C for 1min, and extension at 72°C for 2min and the final step of extension at 72°C for 10min. The thermal cycler program for *FokI* was as follows: 95°C for 6min as initial denaturation, followed by 35 cycles of denaturation at 95°C for 30sec and annealing at 63°C for 30sec, and extension at 72°C for 40sec and the final step of extension at 72°C for 6min. The thermal cycler program for *BsmI* was as follows: 95°C for 2min as initial denaturation, followed by 35 cycles of denaturation at 95°C for 1min and annealing at 63°C for 1min, and extension at 72°C for 90sec and the final step of extension at 72°C for 7min. The primer’s sequence used for the proliferation of VDR gene was:

*FokI*: F5- AGCTGGCCCTGGCACTGACTCTGGCT-3

R 5- ATGGAAACACCTTGCTTCTTCTCCCTC-3

*BsmI*: F 5- CAACCAAGA CTACAAGTACCGCGTCAGTGA-3

R 5- AACCAGCGG GAAGAGGTCAAGGG-3

*ApaI*: F 5- CAGAGCATGGACAGGGAGCAA -3

R 5- GCAACTCCTCATGGCTGAGGTCTC-3

*TaqI*: F 5- CAGAGCATGGACAGGGAGCAA -3

R 5- GCAACTCCTCATGGCTGAGGTCTC-3

**Figure 1:**
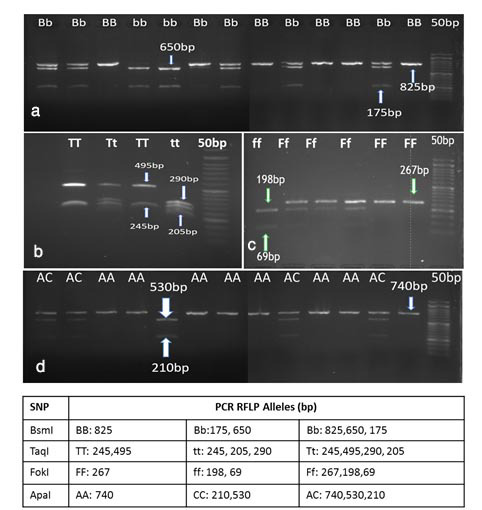
The PCR-RFLP genotyping of VDR SNP in random samples. A-D shows for *BsmI*, *TaqI*, *FokI* and *ApaI* respectively. The molecular marker of 50bp was used, and the table below the figure explains the length (bp) of each allele (homozygous and heterozygous) in the studied SNPs

The genotypes of VDR gene polymorphisms in four regions (*FokI*: rs2228570, *TaqI*: rs731236, *ApaI*: rs7975232, and *BsmI*: rs1544410) were determined by polymerase chain reaction-restriction fragment length polymorphism (PCR-RFLP) [ 20 - 22 ]. All the restriction enzymes were from Thermo Fisher Scientific. For this procedure, the PCR product was cut by restriction enzymes *FokI*, *BsmI*, *ApaI*, and *TaqI*, and then the product was stained with DNA safe stain (Sinaclon, Tehran) on 1.5% agarose gel. After electrophoresis and the appearance of the bands, genotyping was performed based on the cut pattern. After determining the frequency of alleles and genotypes, haplotype analysis was performed using SNPStats. The gel electrophoresis result of the VDR genotyping is shown in Figure 1. 

**Figure 2:**
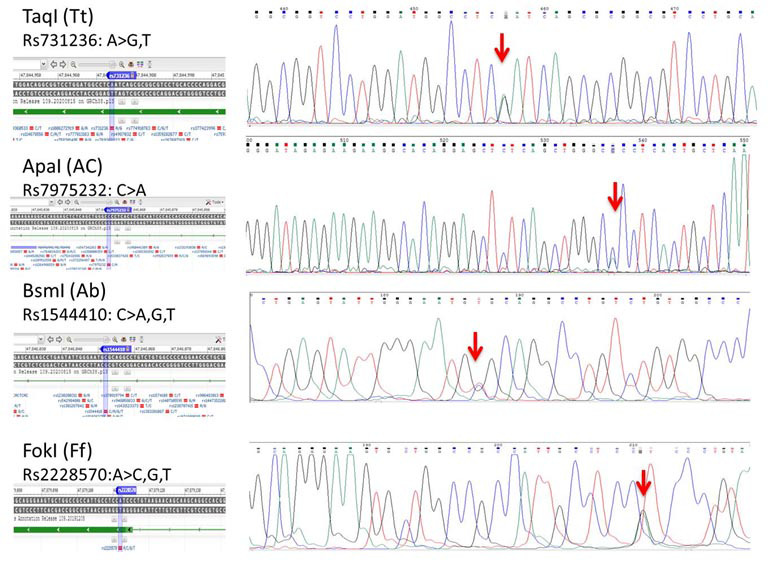
PCR sequencing result of heterozygous samples for each polymorphism to confirm the genotyping method

To confirm the validity of the PCR-RFLP genotyping method, four random PCR sample products (one heterozygous sample for each SNP) sent for direct Sanger sequencing (considered as gold standard method of genotyping), the result of which is reported in Figure 2. 

### Statistical analysis

The collected data were added into the statistical software IBM SPSS Statistics for Windows version 17.0 (IBM Corp, 2011, Armonk, NY: IBM Corp.), which was used for the statistical analysis. Frequency tables were used to describe the results and compare the qualitative variables. The Chi-square test was used for nominal variables analysis. For numeric variables, first of all the normality of distribution was checked then the t-test was used. For SNP analysis the SNPStats web tool was used (https://www.snpstats.net/start.htm). Before SNP analysis the Hardy-Weinberg equilibrium was evaluated first using SNPStats, which showed equilibrant distribution. The odds ratio (OR) and 95% confidence interval (CI) were reported for genes. *p* Values <0.05 were considered statistically significant. 

## Results

A total of 102 patients were included in this study within the age range of 28-58 years (mean of 36.64 years); 51 patients were in the case group, and 51 were in the control group. The sex distribution and the mean age of the groups were not different (*p*> 0.05; Table 1). The mean PD was 4.9±0.87mm in the case group and 1.6± 0.2mm in the control group; the case group had a mean 5.7±0.69mm CAL. Comparing the frequency of alleles of SNP1 (*TaqI*) (*p*= 0.089), SNP2 (*ApaI*) (*p*= 0.481), SNP3 (*BsmI*) (*p*= 0.566), and SNP4 (*FokI*) (*p*= 0.658) showed no difference between the groups (Table 1).

**Table 1:** Comparing the baseline and clinical information of the two groups

Variables		Case group N(%)	Control group N(%)	*p* Value
Sex distribution	M	26 (51)	26 (51)	1*
	F	25 (49)	25 (49)	
Age (years, mean±SD)		36.94±7.55	36.33±7.39	0.341†
Probing pocket depth		6.5±0.87	1.6±0.2	<0.05
Clinical attachment loss		5.7±0.69	-	-
Plaque index		45.34	45.17	>0.05
*TaqI* allele frequency (SNP1)	T	61 (61)	48 (49)	0.089*
	t	39 (39)	50 (51)	
*ApaI* allele frequency (SNP2)	A	57 (59)	63 (64)	0.481*
	C	39 (41)	35 (36)	
*BsmI* allele frequency (SNP3)	B	51 (52)	55 (56)	0.566*
	b	47 (48)	43 (44)	
*FokI* allele frequency (SNP4)	F	80 (80)	79 (77)	0.658*
	f	20 (20)	23 (23)	

*The results of Chi-square test, †the result of the independent samples t-test, M:male, F:female. SNP1(rs731236) alleles:(T:wilde type allele T, t: non wilde type allel C/A), SNP2 (rs7975232) Alleles: (A,C), SNP3 (rs1544410) alleles: B:wilde tpe allele G, b: non wilde type allel A/C/T, SNP4 (rs2228570) alleles: F: wilde type allele T, f: non wilde type allele C/G/A). Notification: VitD receptore gene (ID:7421) is located in reverse direction

Tables 2 [Table T3][Table T4]-5 show the frequency of genotypes; as indicated, the frequency of four inheritance models of dominant, recessive, overdominant, and codominant, were not different between the two study groups in any of the SNPs (*p*> 0.05).

**Table 2:** Studying the difference in the frequency of SNP1 (*TaqI*) genotypes among different genetic models between the two study groups (adjusted by Sex+Age)

Inheritance model	Genotype	Case	Control	OR (95% CI)	*p* Value
Codominant	T/T	19 (38)	15 (30.6)	1.00	0.15
	T/t	23 (46)	18 (36.7)	0.97 (0.38-2.44)	
	t/t	8 (16)	16 (32.6)	2.55 (0.86-7.60)	
Dominant	T/T	19 (38)	15 (30.6)	1.00	0.45
	T/t-t/t	31 (62)	34 (69.4)	1.38 (0.60-3.19)	
Recessive	T/T-T/t	42 (84)	33 (67.3)	1.00	0.052
	t/t	8 (16)	16 (32.6)	2.59 (0.98-6.90)	
Overdominant	T/T-t/t	27 (54)	31 (63.3)	1.00	0.34
	T/t	23 (46)	18 (36.7)	0.67 (0.30-1.52)	

The results of Chi-squared test, SNP1(rs731236) alleles: (T:wilde type allele T, t: non wilde type allel C/A)

**Table 3 T3:** Studying the difference in the frequency of SNP2 (*ApaI*) genotypes among different genetic models between the two study groups (adjusted by sex+age)

Inheritance model	Genotype	Case	Control	OR (95% CI)	*p* Value
Codominant	A/A	15 (31.2%)	20 (40.8%)	1.00	0.61
	A/C	27 (56.2%)	23 (46.9%)	0.64 (0.27-1.54)	
	C/C	6 (12.5%)	6 (12.2%)	0.77 (0.20-2.91)	
Dominant	A/A	15 (31.2%)	20 (40.8%)	1.00	0.34
	A/C-C/C	33	(68.8%) 29	(59.2%) 0.66 (0.29-1.53)	
Recessive	A/A-A/C	42 (87.5%)	43 (87.8%)	1.00	0.99
	C/C	6 (12.5%)	6 (12.2%)	0.99 (0.29-3.39)	
Overdominant	A/A-C/C	21 (43.8%)	26 (53.1%)	1.00	0.36
	A/C	27 (56.2%)	23 (46.9%)	0.68 (0.30-1.54)	

The results of Chi-squared test

**Table 4 T4:** Studying the difference in the frequency of SNP3 (*BsmI*) genotypes among different genetic models between the two study groups (adjusted by sex and age)

Inheritance model	Geno type	Case	Control	OR (95% CI)	*p* Value
Codominant	B/B	13 (26.5)	18 (36.7)	1.00	0.43
	B/b	25 (51)	19 (38.8)	0.54 (0.21-1.38)	
	b/b	11 (22.4)	12 (24.5)	0.77 (0.26-2.28)	
Dominant	B/B	13 (26.5)	18 (36.7)	1.00	0.26
	B/b-b/b	36 (73.5)	31 (63.3)	0.61 (0.26-1.45)	
Recessive	B/B-B/b	38 (77.5)	37 (75.5)	1.00	0.85
	b/b	11 (22.4)	12 (24.5)	1.10 (0.43-2.80)	
Overdominant	B/B-b/b	24 (49)	30 (61.2)	1.00	0.23
	B/b	25 (51)	19 (38.8)	0.61 (0.27-1.36)	

The results of Chi-squared test, SNP3 (rs1544410) alleles: B:wilde type allele G, b: non wilde type allel A/C/T

**Table 5:** Studying the difference in the frequency of SNP4 (*FokI*) genotypes among different genetic models between the two study groups (adjusted by sex and age)

Inheritance model	Genotype	Case	Control	OR ] (95% CI)	*p* Value
Codominant	F/F	31 (62)	30 (58.8)	1.00	0.86
	F/f	18 (36)	19 (37.2)	1.08 (0.47-2.47)	
	f/f	1 (2)	2 (3.9)	1.93 (0.16-23.09)	
Dominant	F/F	31 (62)	30 (58.8)	1.00	0.77
	F/f-f/f	19 (38)	21 (41.2)	1.13 (0.50-2.52)	
Recessive	F/F-F/f	49 (98)	49 (96.1)	1.00	0.61
	f/f	1 (2)	2 (3.9)	1.87 (0.16-21.90)	
Overdominant	F/F-f/f	32 (64)	32 (62.8)	1.00	0.91
	F/f	18 (36)	19 (37.2)	1.05 >(0.46-2.37)	

The results of Chi-squared test, SNP4 (rs2228570) alleles: F: wilde type allele T, f: non wilde type allele C/G/A)

The result of control PCR sequencing indicated the accuracy of the genotyping method (Figure 2). 

**Figure 3:**
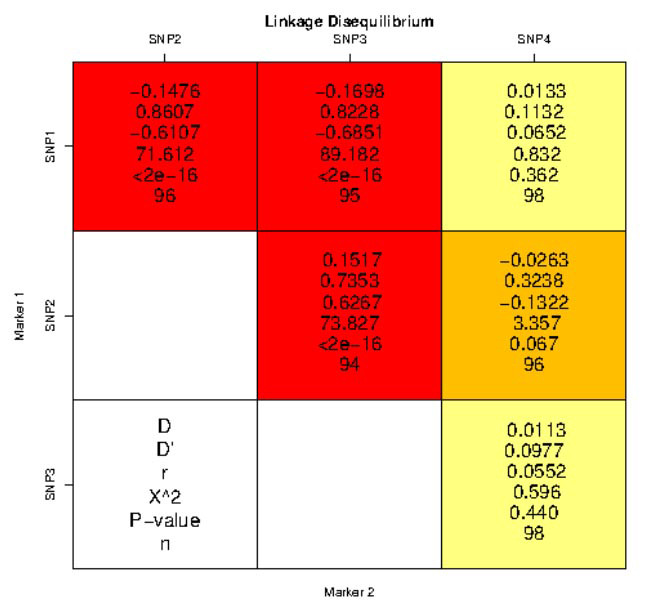
The results of analyzing the linkage between single nucleotide polymorphisms

Multiple SNP analysis showed linkage between SN-Ps 1, 2 and 3 (*TaqI*, *ApaI*, and *BsmI* loci, *p*< 2e16); but not between SNP 1 and 4 (*p*=0.363) or 3 and 4 (*p*= 0.440). The results of the linkage analysis are shown in Figure 3. Accordingly, 12 haplotypes were identified in the study population, the frequency of which is shown in Table 6. The most common haplotype was TCbF in the case group and tABF in the control group, but the frequencies were not different between the two study groups (*p*> 0.05; Table 6). Indeed the global haplotype association was not statistically significant (*p*= 0.28). 

**Table 6:** The frequency of haplotypes in the study population and their association with the disease

Haplotypes	SNP1	SNP2	SNP3	SNP4	Frequency			Frequency Adjusted by sex+age	OR (95%CI) Adjusted by sex+age	*p* Value Adjusted by sex+age
					Total	Case	Control			
1	t	A	B	F	0.2822	0.266	0.333	0.291	1.00	---
2	T	C	b	F	0.2725	0.3006	0.2254	0.267	0.46 (0.16-.32)	0.15
3	t	A	B	f	0.1106	0.086	0.099	0.0999	0.62 (0.13-3.08)	0.56
4	T	A	B	F	0.0862	0.1374	6e-04	0.0684	0.00 (-Inf - Inf)	1
5	T	A	b	F	0.0568	0.0442	0.0841	0.0647	1.26 (0.25-6.43)	0.78
6	T	A	b	f	0.048	0.0317	0.0585	0.0482	2.03 (0.22-18.94)	0.53
7	T	C	B	F	0.0454	0.0264	0.0743	0.0452	1.45 (0.22-9.71)	0.7
8	T	C	b	f	0.0444	0.0689	0.0245	0.0426	0.35 (0.03-4.67)	0.43
9	t	A	b	F	0.0293	0.0261	0.0311	0.0303	0.82 (0.15-4.40)	0.82
10	t	C	B	F	0.015	NA	0.026	0.0181	1.43 (0.03-77.19)	0.86
11	t	C	b	f	0.0095	0.0127	0.0117	0.0246	3.16 (0.00-26072)	0.8
12	T	A	B	f	0	NA	0.0284			

## Discussion

The present study investigated the association between VDR gene (*Fok1*, *Apa1*, *Bsm1*, and Taq1) polymorphisms and severe periodontitis, and the results did not show any statistically significant difference neither in the allele distribution nor in the frequency of the genotypes between the case and control group. Haplotype analysis also showed no difference between the two study groups. These results suggest that VDR gene polymorphisms do not play a role in the etiology of periodontitis in the study population. This issue is of great significance, as by understanding the genetic predisposition of this disease, we may be able to prevent the progression of the primary disease into the advanced chronic form that is associated with poor outcomes [ 23 ]. Looking into the available literature reveals a considerable number of studies addressing this issue; however, the results of these studies are controversial which could be related to the ethnicity variation, different sample size and study design Besides the original research articles comparing the case and control groups, meta-analyses have also been performed [ 16 , 25 , 27 - 29 ]. Nonetheless, each meta-analysis has also reported a different result for the association of VDR gene polymorphisms with periodontitis. In the following, we review the relevant literature available and compare them with the results of the present study.

Although we did not find any significant differences in any of VDR genes (*Fok1*, *Apa1*, *Bsm1*, and Taq1) between two study groups, some have. In a meta-analysis of 15 studies (pooled analysis of 1338 cases vs. 1302 controls), conducted in 2011, periodontitis had a higher frequency of AA genotype of *ApaI* (OR=2.2) in Asians (vs. Caucasians), weakly higher frequency of TT genotype of *TaqI* (OR-1.86) in Asians; while no difference in any genotype of *FokI* [ 16 ] was reported. The mechanism of such an effect has been attributed to the biological role of vitamin D in bone resorption and immune function [ 24 ]. However, the results of the present study revealed no significant association. It should be mentioned that there was not any study from Iran in this meta analysis. In fact, race specific variation in the distribution of genotypes in the VDR gene polymorphism has been demonstrated which can justify these controversial results. Another meta-analysis of 19 case-control studies on the association of VDR gene polymorphisms and chronic or aggressive periodontitis has also suggested a significant association between *TaqI* variants and chronic periodontitis in Asians rather than aggressive periodontitis and whites. They also suggested no association in *BsmI* and *ApaI* polymorphisms with either chronic or aggressive forms of periodontitis, while *FokI* was associated with aggressive periodontitis (OR= 1.58) in Asians [ 25 ]. This is while we did not observe any association with any of the studied polymorphisms of VDR gene. This difference could be related to the influence of race on this association, as previous studies have suggested [ 16 , 25 ]. Another study on Japanese men also detected no association between VDR gene polymorphisms, *ApaI*, *BsmI*, and *FokI* polymorphism with chronic severe periodontitis [ 26 ], which is consistent with the results of the present study. A meta-analysis of the results of 9 trials in a Chinese population suggested no association between VDR *TaqI* polymorphism and the risk of periodontitis [ 27 ]. In a more recent meta-analysis of 19 publications (38 case-control studies), performed in 2017, it was suggested that none of the four VDR gene polymorphisms are associated with chronic periodontitis. Subgroup analysis, stratified by ethnicity, showed an association of *BsmI* polymorphism with chronic periodontitis in the Caucasian subgroup under the allele model (A vs. G, OR=1.75), but not in the Asian, mixed, and African populations. When stratified by Hardy-Weinberg equilibrium, no association was observed. The studies included in this meta-analysis were from multiple races and also used different genotyping methods (PCR, PCR-RFLP and sequencing) and finally, the author concluded that the VDR gene might not be associated with the risk of chronic periodontitis in the overall population [ 28 ]. These results are consistent with the results of the present study and suggest that the genetic predisposition to periodontitis differs based on the race/ethnicity of the study population. In 2019, another meta-analysis pooled data of 34 relevant studies (3848 patients with periodontitis vs. 3470 controls), and the results suggested a link between VDR Bs-mI (OR=0.72) and *FokI* (OR=1.46) in the general population; when stratified by race, *FokI* remained significant in the Chinese population (OR=1.8) and *TaqI* in the Caucasian population (OR=0.52); when stratified by type of periodontitis (OR=2.02 for aggressive periodontitis) [ 29 ]. These results are inconsistent with the results of the present study. These differences could be attributed to the variations in study design, sample sizes, and heterogeneous populations.

As presented above, the association of VDR gene polymorphisms with periodontitis is a controversial issue and varies based on several factors; including sample size, genotyping method and ethnicity. Our study population was Iranian and thus may be different from the results of studies performed in other countries; however, only a few studies have been performed on the Iranian population. One Iranian study compared 69 patients with chronic periodontitis with 78 matched healthy controls and showed no association between chronic periodontitis and *TaqI* and *ApaI* alleles or genotypes [ 30 ], which is in line with the results of the present study. Interestingly, they suggested an association of *ApaI* with different disease severities [ 30 ], which, similar to the results of previous studies, suggests the role of this gene in more severe diseases. In another study in Tehran, Iran, 50 patients with periodontitis were compared with 50 controls, and the results showed no association between polymorphism of *ApaI* and periodontitis [ 31 ]. These results are in line with that of the present study for *ApaI* genotype and allele, although we evaluated three others, as well. Similar to the results of the present study, they have also reported the frequency of alleles in the two groups (AA, AC, and CC Genotypes were 50%, 28%, and 22% in cases and 52%, 32% and 16% in controls), which seemed to be different without statistical significance. Apparently, the frequency of genotypes is also different in different races/ ethnicities. In a study in Indonesia, it was found that Tt and tt genotypes of *TaqI* are lower than TT genotype both in patients with periodontitis and the control group [ 32 ]. Others have also reported similar results, indicating Tt and tt genotypes is only in 2% of Asians, 4% of Chinese [ 33 ], 5% of African Americans, 11% of Japanese [ 34 ], and 17% of Caucasians [ 35 ].

The present study is one of the few studies addressing this issue in this specific population. However, the sample size of this study was limited, and participants were selected from one dental center in the north of Iran. Accordingly, the results may not be generalizable to the whole population of this country. Nevertheless, the results can be used by physicians, dentists, and policy-makers until a more comprehensive or population-based study becomes available. It should be noted that there may be some factors confounding the results of the present study, such as the oral health/hygiene of the individuals, which we considered and homogenized in the study population using the plaque index. We also included only non-smokers in this study to omit the effect of smoking; however, there may be other factors, as well, such as lifestyle or environmental factors, which we were not aware of and did not control in this study, but we assume that the randomized enrollment of participants into the study and comparison between two matched groups reduce the chance of the effect of confounders on the results of this study. 

Altogether, according the literature review, there is inconsistent findings regarding the association between vitamin D receptor gene polymorphisms (*BsmI*, *FokI*, *ApaI*, and *TaqI*) and periodontitis. Some research indicates significant associations, while others report no correlation, which could be likely due to differences in sample sizes, population diversity, and methodological approaches. Moreover, factors such as diet, lifestyle, and exposure to pathogens can interact with genetic predispositions, complicating the interpretation of genetic associations with periodontitis. 

## Conclusion

Our findings showed no association between VDR gene polymorphisms (SNP1 (*TaqI*), SNP2 and periodontitis in a population from Northeast of Iran. Considering the controversies in the previous studies and the limitations of the present study, more comprehensive studies are required for a definite conclusion about VDR-related genetic predisposition in periodontitis
